# Storage conditions affect the composition of the lyophilized secretome of multipotent mesenchymal stromal cells

**DOI:** 10.1038/s41598-024-60787-z

**Published:** 2024-05-03

**Authors:** Olena Rogulska, Irena Vackova, Simon Prazak, Karolina Turnovcova, Sarka Kubinova, Lucie Bacakova, Pavla Jendelova, Yuriy Petrenko

**Affiliations:** 1 Laboratory of Biomaterials and Tissue Engineering, Institute of Physiology of the Czech Academy of Sciences, Videnska 1083, 14200 Prague, Czech Republic; 2 Department of Neuroregeneration, Institute of Experimental Medicine of the Czech Academy of Sciences, Videnska 1083, 14200 Prague, Czech Republic; 3 Department of Optical and Biophysical Systems, Institute of Physics of the Czech Academy of Sciences, Na Slovance 1999/2, 18221 Prague, Czech Republic

**Keywords:** Cell biology, Regenerative medicine, Stem-cell biotechnology

## Abstract

The widespread use of multipotent mesenchymal stromal cell-derived secretome (MSC-sec) requires optimal preservation methods. Lyophilization offers benefits like concentrating the secretome, reducing the storage volume, and making storage conditions more flexible. This study evaluated the influence of storage duration and temperature on lyophilized MSC-sec. The conditioned medium from Wharton’s jelly MSCs was stored at – 80 °C or lyophilized with or without trehalose. Lyophilized formulations were kept at – 80 °C, − 20 °C, 4 °C, or room temperature (RT) for 3 and 30 months. After storage and reconstitution, the levels of growth factors and cytokines were assessed using multiplex assay. The storage of lyophilized MSC-sec at – 80 °C ensured biomolecule preservation for 3 and 30 months. Following 3 month storage at 4 °C and RT, a notable decrease occurred in BDNF, bNGF, and sVCAM-1 levels. Prolonged 30 month storage at the same temperatures significantly reduced BDNF, bNGF, VEGF-A, IL-6, and sVCAM-1, while storage at – 20 °C decreased BDNF, bNGF, and VEGF- A levels. Trehalose supplementation of MSC-sec improved the outcome during storage at 4 °C and RT. Proper storage conditions were crucial for the preservation of lyophilized MSC-sec composition. Short-term storage at various temperatures maintained over 60% of the studied growth factors and cytokines; long-term preservation was only adequate at −80 °C.

## Introduction

The shift from the direct use of multipotent mesenchymal stromal cells (MSCs) to their bioactive products has become more evident in cell therapy over the last decade^1^. Key complementary mechanisms behind the therapeutic action of MSCs include paracrine action of their secretome components, intercellular mitochondria transport, and the exchange of extracellular vesicles containing microRNA, lipids, and metabolites^2,3^.

Currently, it has been proven that a cocktail of trophic factors and inflammatory modulators released by MSCs exhibits potent therapeutic efficacy in numerous preclinical models^4–6^ and clinical trials for the treatment of alopecia^7,8^, post-acne scars^9^, chronic plantar ulcers in leprosy^10^, for the wound healing^11^ and bone regeneration^12^. Compared to classic whole-cell MSC infusions/injections, the use of MSC secretome (MSC-sec) as a cell-derived product has benefits in terms of safety, reproducibility, and the possibility of technically facile and economical mass production^13^.

The broad translation of MSC-sec into research and future clinical practice requires the development of specific preservation conditions, which ensure the stability of the samples during long-term storage. The storage of proteins at/below −80 °C before analysis or further applications is considered a golden standard of the research- and clinical-grade routine, aiming to preserve samples with high quality. As an alternative, the lyophilization (or freeze-drying) method may provide the possibility to form concentrated MSC-sec stocks that do not require storage in liquid nitrogen, allowing large clinical trials or scientific research in facilities lacking specialized cell culture equipment^14^.

It has been shown that lyophilization does not significantly alter the stability of secretome proteins and lipids^15,16^. In the study of Bari et al.^17^, a proteomic analysis helped to identify more than 349 distinct proteins in lyophilized batches of MSC-sec obtained using a validated good manufacturing practice (GMP)-compliant process. This set of proteins, which are involved in immune response, reaction to stress, motility and metabolism, represents classic components of fresh MSC-sec collected without any additional manipulations. A direct comparison between lyophilized and non-lyophilized samples of MSC-sec showed similar levels of growth factors, such as vascular endothelial growth factor-A (VEGF-A), fibroblast growth factor-2 (FGF-2), transforming growth factor-beta1 (TGF-β1), insulin-like growth factor-1 (IGF‐1), chemokines and cytokines, such as C-X-C motif chemokine ligand 10 (CXCL10) and interleukin-6 (IL-6)^14,16,18^.

Preservation of the therapeutic potency of MSC-sec after lyophilization has been shown in numerous preclinical models of dry eye syndrome, acute lung injury, corneal epithelial and skin wound healing, and many others^15,19–21^, confirming high research and clinical potential of lyophilized MSC-sec.

Nevertheless, little is known about the optimal conditions for storage of ready-to-use MSC-sec preparations. Regarding temperature regimes, no standardized approach is currently available, and according to reports freeze-dried conditioned medium (CM) has been stored at − 80 °C, − 20 °C and even at + 4 °C until use^17,18,21^. Most papers lack information about storage duration, and the small number of examples showing therapeutic activity of lyophilized secretome after 5–6 months of storage can be considered as exceptions^17,22^.

The information available from experiments with samples of plasma, isolated proteins or supernatants makes it reasonable to suggest that the storage conditions determine the stability of MSC-sec components^23,24^. For example, storage at room temperature has been proven to affect the concentration of lipoproteins and choline compounds in freeze-dried blood plasma, while storage at − 20 °C for more than 7 days induced protein aggregation^24^. Laggner et al.^22^ reported an alteration in the activity of several biomolecules derived from stressed peripheral blood mononuclear cell secretome, although the physicochemical properties remained consistently stable for up to 6 months when storing lyophilizates between − 20 °C and + 25 °C.

It has been shown that the supplementation of different protein formulations, including CM, by sugar-based excipients such as sucrose, trehalose and mannitol enhances the stability of the biomolecules during freezing and lyophilisation^17,25,26^. However, the effect of such supplementation during the storage of lyophilized preparations at different temperatures and/or storage periods has not yet been completely revealed.

In the present study, we aimed to characterize changes in the concentration of growth factors and cytokines in lyophilized MSC-sec after storage during a short-term (3 months) and a long-term period (30 months) at different temperatures in the presence or absence of trehalose as a stabilizing supplement.

We used human umbilical cord Wharton’s jelly MSCs (WJ-MSCs) to obtain MSC-sec. The umbilical cord, often discarded postpartum, offers an ethically non-controversial and readily available source of MSCs. Previously, we have confirmed a remarkably higher proliferation capacity of WJ-MSCs than MSCs derived from human adult bone marrow and adipose tissue^27^. WJ-MSC-derived secretome contained higher amounts of hepatocyte growth factor (HGF), FGF-2, brain-derived neurotrophic factor (BDNF), and beta-nerve growth factor (bNGF) than other cell types assessed. It ensured the neuroprotective/neurotrophic effect in co-culture models in vitro.

In this follow-up study, we lyophilized the MSC-sec obtained from WJ-MSCs and focused on the effect of storage conditions on the stability of its components, which are crucial for therapeutic effects.

## Results

### The composition of the lyophilized MSC-sec after 3 months of storage at different temperatures

To reveal the effect of storage conditions on the lyophilized MSC-sec composition, the freeze-dried CM of WJ-MSCs was stored at four temperatures (− 80 °C, −20 °C, 4 °C and RT) for 3 and 30 months. The non-lyophilized medium stored at – 80 °C served as a control group, corresponding to classical widely accepted storage conditions. Following the storage period, the lyophilized CM was reconstituted, and 7 growth factors and cytokines were assessed by a multiplex assay. Previously, it had been shown that the Luminex bead arrays display variability between production batches, particularly at the detection limits, thereby hindering the comparison of results between assays. Therefore, in this study, we related the concentration of growth factors and cytokines in lyophilized MSC-sec stored at different temperatures to the level of the same analytes in a sample of CM stored for the same time in the liquid state at – 80 °C. This ensured a reliable control, which minimized the inaccuracies associated with the batch-to-batch variabilities of different multiplex assays analysed after 3 and 30 months of storage.

**Figure 1:**
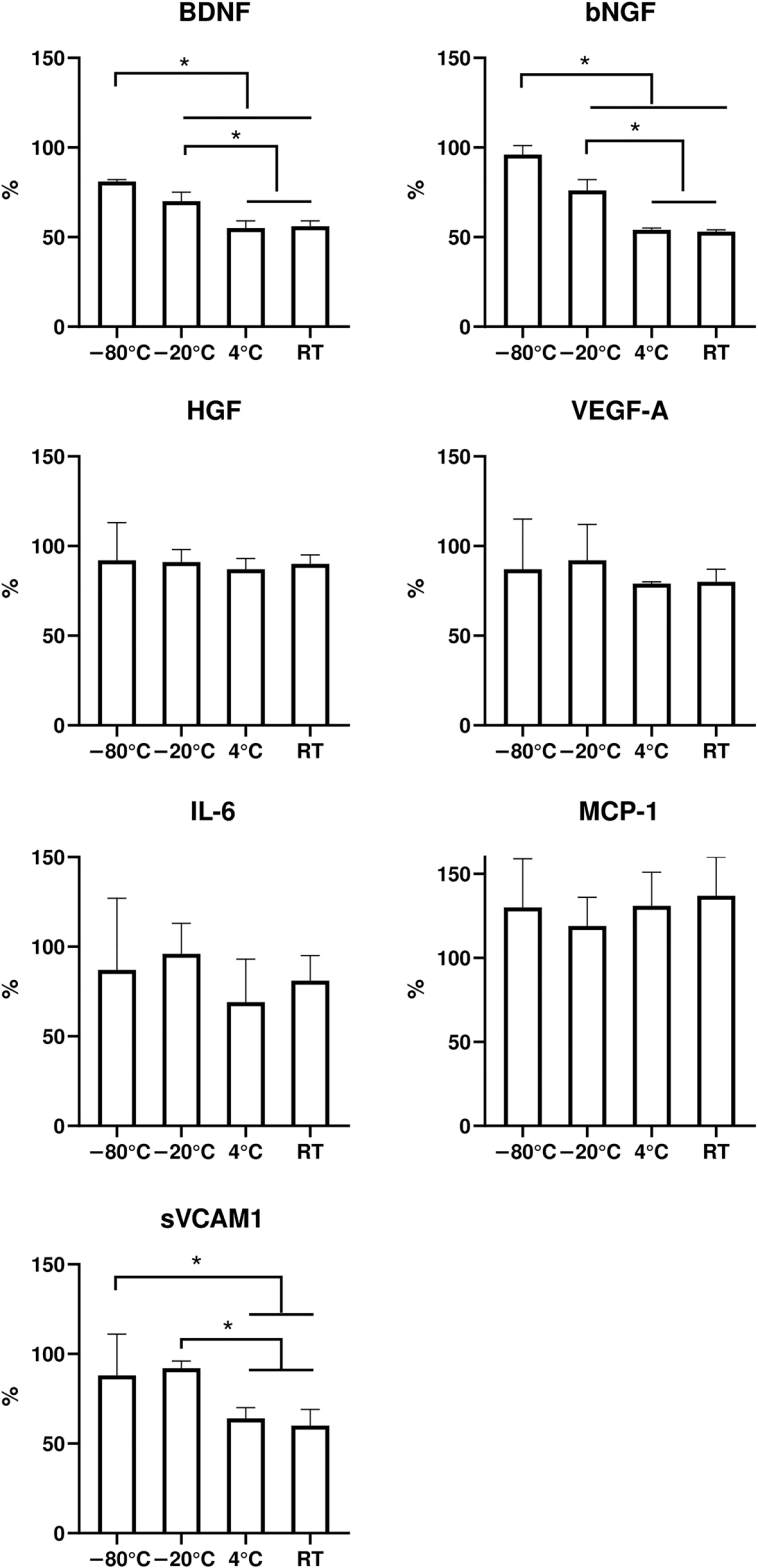
Preservation of the growth factors and cytokines in the lyophilized MSC-derived conditioned medium after 3 months of storage at different temperatures (N = 3). Data is presented as Median (Q1; Q3), * - p < 0.05. The values are related to non-lyophilized MSC-sec stored for the same time (3 months) in a frozen state at − 80 °C.

Figure 1 shows the preservation of selected components of lyophilized MSC-sec after 3 months of storage at different temperatures. After storage at – 80 °C, the content of all evaluated components of lyophilized MSC-sec was preserved at high levels, reaching more than 80% of the values obtained from non-lyophilized CM. No significant differences in the content of HGF, IL-6, VEGF-A and monocyte chemoattractant protein-1 (MCP-1) were detected for any storage temperature (Fig. 1). At – 20 °C, we revealed a significant decrease in neurotrophic factors (BDNF and bNGF) compared to – 80 °C storage. In this case, the median quantity of BDNF comprised 70% (69.8%; 74.8%) and bNGF 76.3% (75.2%; 79%). A more prominent decrease in the levels of several MSC-sec components was indicated after 3 months of storage at positive temperatures (4 °C and RT). In contrast to storage at – 80 °C, a significant reduction in the content of BDNF, bNGF and soluble vascular cell adhesion molecule-1 (sVCAM-1) was observed after storage at both 4 °C and RT. A decrease of around 25% compared to the – 80 °C group was observed in the median content of BDNF, 45% in bNGF and 30% in sVCAM-1 (Fig. 1). Moreover, these values were significantly lower than those obtained after – 20 °C storage. There were no significant differences between the levels of these MSC-sec components after storage at 4 °C and RT.

**Figure 2:**
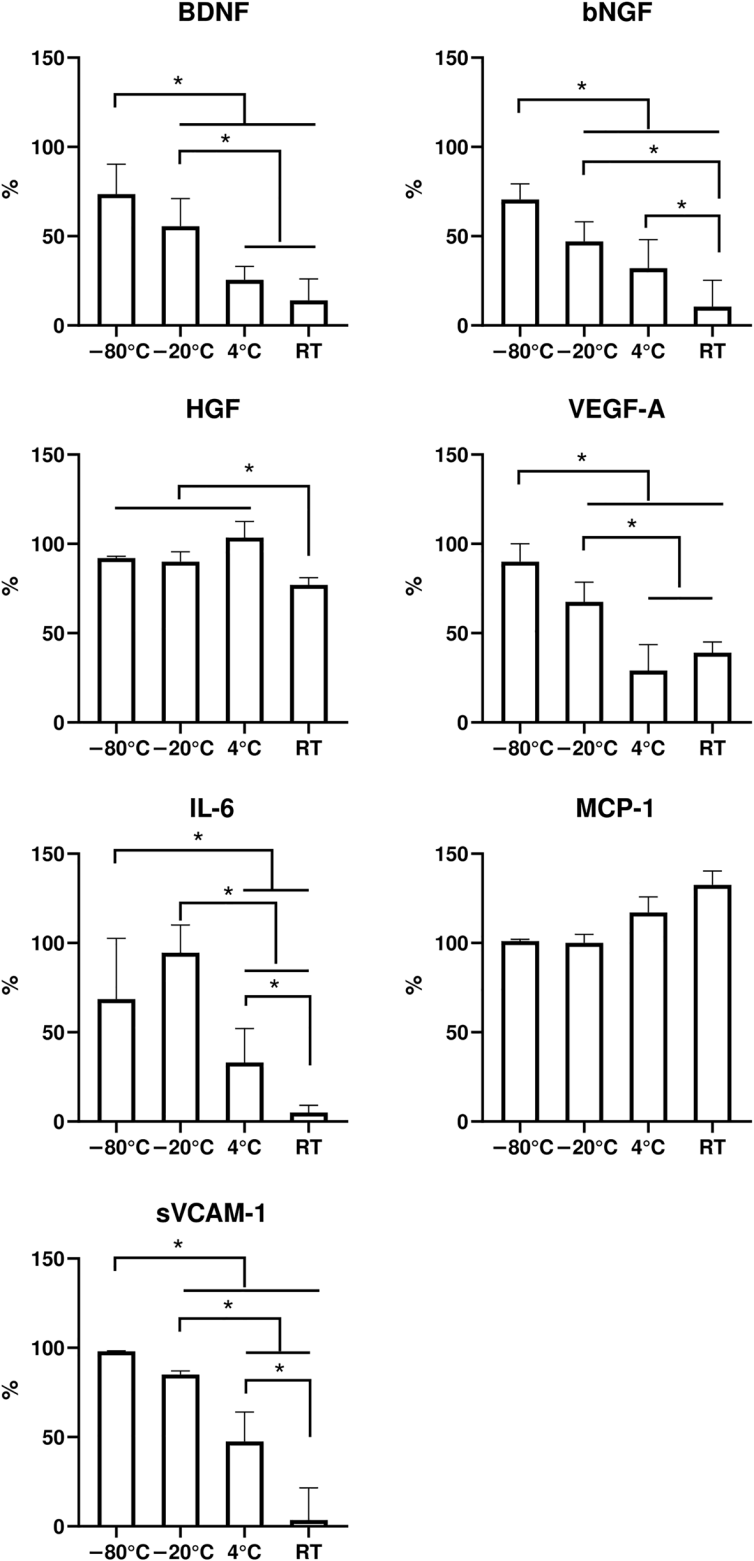
The preservation of growth factors and cytokines in the lyophilized MSC-derived conditioned medium after 30 months of storage at different temperatures (N = 3). Data is presented as Median (Q1; Q3). * - p < 0.05. The values are related to non-lyophilized MSC-sec stored for the same time (30 months) in a frozen state at − 80 °C.

The presented data show that the storage of the lyophilized CM at ultra-low temperatures (− 80 °C) did not result in significant changes of MSC-sec composition, compared to frozen control. This confirms the suitability of lyophilization as a method for secretome preservation. However, the preservation of the secretome cocktail was affected by the storage temperature. We then evaluated the preservation of the lyophilized MSC-sec composition after 2.5 years (30 months) of storage at different temperatures (Fig. 2).

### The composition of the lyophilized MSC-sec after 30 months of storage at different temperatures

Maintaining lyophilized CM samples at – 80 °C did not cause significant changes in the MSC-sec composition, and the levels of growth factors and cytokines did not drop below 70% of the levels in the non-lyophilized frozen control. Similar to 3 months of storage, no significant decrease in MCP-1 was detected after 30 months, evidencing high resistance of this chemokine to harmful factors associated with lyophilization and prolonged storage. The levels of HGF in lyophilized MSC-sec stayed stable after 30 months of storage at – 80 °C, − 20 °C, and 4 °C; however, a significant decrease was detected after the maintenance of CM at RT. In this case, the levels remained at around 70% (Fig. 2).

In contrast to the 3 month storage period, the level of VEGF-A in the lyophilized CM decreased significantly after 30 months of storage. A loss in the median quantity of VEGF-A was detected after storage at – 20 °C, comprising 68% (58%; 75%). An even more pronounced decrease was observed in secretome stored at positive temperatures. In this case, the level of VEGF-A comprised 29% (27%; 42%) for 4 °C and 39% (37%; 43%) for RT. Both values were significantly different compared to the – 20 °C storage group.

The content of neurotrophic factors (BDNF and bNGF) in the lyophilized MSC-sec decreased after 30 months of storage at – 20 °C, 4 °C and RT. The median of 56% (51%; 68%) of BDNF and 47% (35%; 54%) of bNGF were preserved at – 20 °C, which was significantly lower compared to the – 80 °C group. At higher temperatures, the content of neurotrophic factors did not exceed 40%. Here, the median quantity of BDNF in lyophilized MSC-sec stored at 4 °C comprised 25% (24%; 34%), whereas at RT the BDNF in the conditioned medium was almost lost, being only 14% (10%; 30%). The drop in bNGF content had a similar tendency: 32% (26%; 46%) of median bNGF was detected after 30 months of storage at 4 °C, while at RT the median bNGF level comprised only 11% (7%; 28%). Moreover, the bNGF levels obtained after storage of lyophilized MSC-sec at RT were significantly lower compared to the 4 °C group. Similar dynamics were observed in the sVCAM-1 content after storage. At −20°C, the quantity of this adhesion molecule decreased to 85% level compared to non-lyophilized frozen control, but at 4 °C, the quantity was preserved only at 47% (32%; 64%) and was lost after storage at RT, being only 3% (1%; 17%). The IL-6 preservation efficiency was also closely related to the storage temperature. Although no prominent decrease was detected after storage of the secretome at – 20 °C, a remarkable drop was detected at positive temperatures. At 4 °C, the level of IL-6 comprised 33% (31%; 52%), while at RT, it remained at only 5% (4%; 8%).

As expected, we found that prolonged (30-month) storage of MSC-sec in non-optimal conditions led to a significant change in the overall paracrine cocktail composition. Even when stored at – 20 °C, we detected lower amounts of neurotrophic factors (BDNF and bNGF). Positive temperatures remarkably affected the lyophilized MSC-sec composition, sometimes leading to a complete loss of the secretome molecules at RT (e.g. BDNF, bNGF, IL-6 and sVCAM-1). To stabilize the biomolecules and protect them from lyophilization-associated stresses, we supplemented MSC-sec, in parallel studies, with 40 mM of trehalose, a known stabilizer of proteins during freezing and/or drying, prior to lyophilization.

### The effect of trehalose as a supplement to the lyophilized formulation on the secretome composition after storage

The addition of 40 mM trehalose had no significant effect on the preservation of lyophilized MSC-sec stored at all monitored temperatures for 3 months. Similarly, no remarkable differences were detected when the secretome was stored at – 20 °C or – 80 °C for 30 months. Nevertheless, after prolonged (30 month) storage at 4°C and RT, a significant positive effect of the supplement was detected when evaluating the content of BDNF, bNGF, IL-6 and sVCAM-1 (Table 1).

**Table 1:** The preservation of growth factors and cytokines in the MSC-derived CM after lyophilization in the presence or absence of trehalose and following 30 months of storage at positive temperatures.

	4 °C		RT	
	No trehalose	With trehalose	No trehalose	With trehalose
BDNF	25% (24%; 34%)	54% (48%; 69%)*	14% (10%; 30%)	38% (28%; 39%)*
bNGF	32% (26%; 46%)	49% (45%; 74%)*	11% (7%; 22%)	33% (25%; 46%)*
IL-6	33% (31%; 52%)	69% (57%; 79%)*	5% (4%; 8%)	11% (9%; 12%)*
sVCAM-1	47% (32%; 64%)	59% (57%; 64%)*	3% (1%; 17%)	29% (17%; 34%)*

*The values are significantly (p < 0.05) higher compared to the “no trehalose” group. The values are related to non-lyophilized MSC-sec stored for the same time (30 months) in a liquid state at − 80 °C.

Improved BDNF and IL-6 preservation was achieved by trehalose supplementation when lyophilized MSC-sec was stored at 4 °C and at RT, though the content of IL-6 in secretome stored at RT for 30 months was still low and comprised only 11% (9%; 12%). Significantly higher quantities of bNGF and sVCAM-1 were detected in the secretome lyophilized in the presence of trehalose after storage at both positive temperatures (Table 1). Here, after storage at RT, the content of sVCAM-1 was almost 10 times higher compared to the trehalose-free formulation. Therefore, the addition of 40 mM trehalose into the conditioned medium prior to lyophilization improved the preservation of the overall composition of the secretome stored at 4 °C and RT.

## Discussion

In the present study, we have assessed the effect of storage conditions on the levels of growth factors and cytokines in lyophilized conditioned media. It is known that even in a dried state, the stability of biomolecules declines with an increase in storage temperature^22,28^. At the same time, there is a lack of studies concerning the interplay between lyophilized secretome stability and storage duration. Short-term stability is essential to overcome batch-to-batch variability in laboratory settings, while long-term stability should be considered for establishing cryobanks of ready-to-use samples for retrospective or longitudinal studies^29^.

In this research, we focused on the analysis of a defined set of growth factors (BDNF, bNGF, HGF, VEGF-A, IL-6, MCP-1, sVCAM-1) within the MSC-sec, which was previously confirmed to have a neurotrophic and neuroprotective activity in in vitro co-culture models^27^. We believe that the assessment of the preservation of these molecules can potentially be used as a quality control parameter for lyophilized MSC-sec formulations.

**Figure 3:**
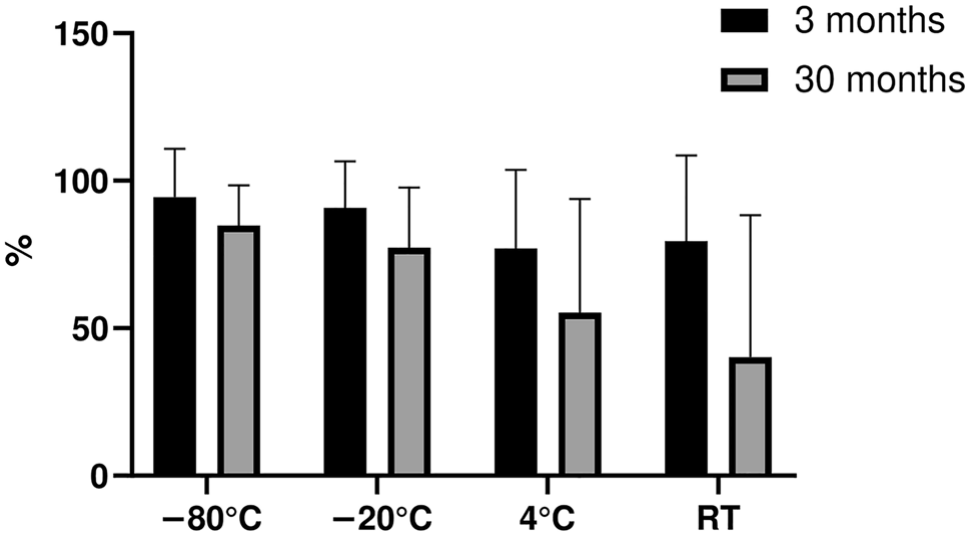
The preservation levels of the whole lyophilized MSC-sec cocktail, determined as the median of the preservation values for each of the MSC-sec components. The values are related to non-lyophilized MSC-sec stored for the same time in a frozen state at − 80 °C.

Our results indicate that 3 month short-term storage at temperatures higher than − 80 °C resulted in a decrease in the content of BDNF and bNGF. However, the median preservation level of the whole MSC-sec cocktail remained at 65–80%, depending on the storage temperature (Fig. 1, Fig. 3).

Long-term storage (30 months) deteriorated the stability of proteins in lyophilized MSC-sec more intensively than short-term storage (3 months) (Fig. 2, Fig. 3). However, the severity of the detrimental effects differed depending on the storage temperature and the individual molecules. We demonstrated a remarkable drop in the content of BDNF, bNGF, VEGF-A, IL-6, and sVCAM-1 after storage of lyophilized MSC-sec at positive temperatures (i.e. 4 °C and RT). A less severe decrease in the levels of growth factors and cytokines within lyophilized MSC-sec stored at − 20 °C was also detected, whereas in the case of storage at − 80 °C, the composition of the secretome cocktail was mainly preserved. The molecular mobility and the velocity of the reactions, including damaging reactions, are drastically slowed down at negative temperatures. In our experiments the highest stability of the cytokine level was achieved at − 20 °C and − 80 °C. However, some of the measured cytokines in MSC-sec could not remain stable over a prolonged time (more than 2 years), which is in line with the data shown on frozen plasma samples^29^.

It can be suggested that the extent of damage associated with lyophilization, freezing, and storage depends on the abundance of reactive side chains in the structure of different cytokines. For example, methionyl residues and free sulfhydryl groups may be oxidized during long-term storage to the sulfoxide and disulfide forms, correspondingly (reviewed in^30^). Sulfhydryl groups also participate in the formation of intra- and inter-chain disulfide bonds, which in turn can cause the formation of non-natural polypeptide dimers and trimers, leading to subsequent aggregation of proteins. In addition to oxidation, acid–base-driven hydrolysis reactions might result in changes in metabolite concentrations, as was shown for plasma samples^24^. Mentioned modifications of amino acid residues can cause alteration of the secondary, tertiary, and quaternary protein structure, while changes in pH and temperature observed during freeze-drying and storage can trigger denaturation^30^. The accumulation of reactive oxygen species should be mentioned among the other damaging factors that interfere with the structural integrity and the therapeutic activity of stored MSC-sec components^31^.

In attempts to stabilize biomolecules and protect them from lyophilization-linked stresses, many additives have been tried, with sugars and their derivatives being the most effective against dehydration, freezing, and high osmolality-induced damage^30^. It was shown that mannitol inclusion into the composition of MSC-sec preserved the integrity and the morphology of extracellular vesicles in lyophilized batches. In addition, such lyophilized media, stored at – 20 °C before use, protected cells from the oxidative stress damages induced by hydrogen peroxide^17^. Likewise, sucrose was proven to be effective for preserving the antigen-binding activity of freeze-dried plasma-derived immunoglobulin G when stored at − 20 °C and 20 °C^26^. A comparable analysis of the efficiency of glucose, sucrose, and trehalose in diminishing protein aggregation demonstrated equal potential. However, only trehalose reduced the formation of ROS and protein oxidation products during storage^31^.

In our study, we chose trehalose as a protective agent since it has a higher glass transition temperature than most sugars, which makes it suitable for both low-temperature and room-temperature storage. Indeed, the beneficial effect of trehalose on the maintenance of cytokines and growth factor levels was the most prominent at 4 °C and RT. In some cases, due to the small sample size and high donor-to-donor variability in individual protein levels of CM, the stabilization capacity of trehalose was not high enough to reach statistical significance, although a positive trend was observed. The negligible effect of trehalose under low-temperature storage in the current study might be potentially improved by increasing its concentration since it has recently been shown that sugars reduce lyophilization-induced protein aggregation in a dose-dependent manner^31^. Presumable mechanisms behind observed stabilization include the replacement of hydrogen bonds when the hydration shell of the biomolecules is removed and the formation of a highly viscous glassy matrix that reduces the rate of damaging reactions and diffusion of crystals or oxidation products^30,31^.

To sum up, lyophilization may be a valuable strategy for producing an enriched formulation of MSC-sec, which can be easily manipulated, stored, shipped, and used. However, research teams must carefully consider which storage options will lead to the required outcomes. The results obtained in our study show that the maximum stability of the lyophilized MSC-sec composition was obtained during storage at – 20 °C (for short-term maintenance) and at – 80 °C (for both short-term and long-term preservation). At 4 °C and RT, the stability of the majority of MSC-sec biomolecules was significantly reduced. The addition of trehalose into the CM formulation before lyophilization increased the preservation levels of several proteins after a 30 month storage period. It should be mentioned that keeping a stable negative temperature during storage is critical, frozen samples should be thawed only once, immediately before use. Multiple freeze–thaw cycles have been proven to result in a considerable loss of biomolecule activity, regardless of the initial storage temperature of the samples^32,33^.

The study presented here has offered observations that are of practical utility. It has also highlighted the importance of detailed sample history tracking, particularly the storage time and temperature of the lyophilized product. This is indispensable for the appropriate interpretation of the results and for assessing clinical efficacy.

### Limitations of the study

Although we attempted to mimic the clinically relevant conditions of WJ-MSC processing by applying xeno-free culture conditions, the current study cannot serve as a guideline for the GMP-compliant production of therapeutic preparation. To reach the commercial product or clinical formulation standards, many additional essential characteristics of the secretome preparations, such as physicochemical parameters, nanoparticle measurement, purity, and thorough potency tests, should be defined^22,34^.

This study lacks data on the fresh MSC-sec composition, and the changes in the MSC-sec after storage were related to the non-lyophilized samples stored at – 80 °C, which is widely applied in research-grade routine. This limitation arises due to logistical constraints related to the collection of material from different donors over varying time periods. To maintain consistency in secretome evaluation, we opted to analyze all samples using one batch of assay kits for Luminex bead arrays. It necessitated the use of non-lyophilized samples stored at – 80 °C as controls, thereby precluding the assessment of changes in secretome composition immediately post-collection.

Nevertheless, considering a lack of long-term studies showing the effect of storage conditions of lyophilized preparations on the stability of the protein components, our research aimed to highlight the importance of choosing the correct storage conditions in further research and pharmaceutical preparations.

## Materials and methods

### Multipotent mesenchymal stromal cell culture

The study was approved by the Ethics Committee of the Institute of Experimental Medicine of the Czech Academy of Sciences. Wharton’s jelly-derived MSCs were isolated from human umbilical cords according to proper ethical guidelines and were cultured in a xeno-free complete culture medium consisting of α-minimal essential medium (αMEM; LONZA, Basel, Switzerland), 5% pooled human platelet lysate (PL; Bioinova, Ltd., Prague, Czech Republic), and 10 µg/mL gentamicin (Sandoz, Holzkirchen, Germany) as described in our previous publications^27,35^. Cells were cultured at 37 °C in a humidified atmosphere containing 5% CO_2_ with regular media changes twice a week.

### Preparation of MSC-derived secretome, lyophilization and storage

WJ-MSCs from three donors at passage 3 were seeded separately into Nunc™ culture flasks (Nunc, Roskilde, Denmark) at a density 3 × 10^3^/cm^2^ and were cultured till 90% confluence. One day before CM harvesting, the medium was completely changed, and only one half of the original medium volume was added to concentrate the growth factors and other analytes in the CM. In the following 24 h, the secretome was collected, centrifuged at 1500 rpm for 10 min and filtered through a 0.22 µm filter (TPP, Trasadingen, Switzerland). Concurrently, WJ-MSCs were harvested to assess the number of cells producing CM.

The CM from each sample was divided into 9 groups (100 µl CM per sample). One CM sample was fast frozen and stored at − 80 °C (control group). The remaining 8 samples were intended for lyophilization. One half of these CM samples (4 samples) was lyophilized without additional manipulations, whereas the other half (4 samples) was supplemented with trehalose prior to lyophilization. For this, 400 mM trehalose solution was added (1:10) to obtain a final trehalose concentration of 40 mM. The CM samples for lyophilization were fast frozen at − 80 °C and were freeze-dried using a FreeZone freeze dryer (Labconco, Kansas City, MO, USA). The freeze-dried samples were stored at − 80 °C, − 20 °C, + 4 °C and at room temperature (RT) for 3 and 30 months. All samples were prepared in duplicates.

### Reconstitution of lyophilized CM and multiplex analysis

Following storage under different conditions for 3 and 30 months, all samples were reconstituted with 100 µl demineralized H_2_O, and the concentration of selected growth factors and cytokines, namely HGF, MCP-1, BDNF, bNGF, sVCAM-1, IL-6, and VEGF-A in CM was assessed by Luminex^®^-based multiplex ProcartaPlex^®^ Immunoassay (Thermo Fisher Scientific, Waltham, MA, USA). The measurement was performed on a Bio-Plex 200 Instrument (Bio-Rad, Prague, Czech Republic) according to the manufacturer’s instructions. All samples were analysed in duplicate. The cytokine and growth factor concentrations (pg/ml or ng/ml) were derived from the measured Mean fluorescence intensity (MFI) using fitted standard curves.

### Statistical analysis

To analyse the obtained data for normality, a Shapiro–Wilk test was performed. Data were expressed as the median and the interquartile range (Q1; Q3) and were compared using the Kruskal–Wallis ANOVA test for multiple comparisons between the groups. Cut-off p-values less than p < 0.05 were considered statistically significant. For the statistical analysis, the Past v. 3.0 software package was used.

### Ethics approval and consent to participate

All experimental procedures were conducted in accordance with the Declaration of Helsinki, as well as relevant ethical guidelines and regulations. The study was approved under the project “Safety and efficacy evaluation of allogeneic mesenchymal stem cells derived from umbilical cord tissue in repair of cartilage defects: preclinical study on animal model” by the Ethics Committee of the Institute of Experimental Medicine of the Czech Academy of Sciences, No. 2017/07 on 9.07.2017. The informed consent for umbilical cord tissue collection was obtained from all subjects and/or their legal guardian(s).

## Data Availability

The data generated during the current study are available from the corresponding authors upon reasonable requests.

## Abbreviations

BDNF: Brain-derived neurotrophic factor
bNGF: Beta-nerve growth factor
CM: Conditioned medium
CXCL10: C-X-C motif chemokine ligand 10
FGF-2: Fibroblast growth factor-2
GMP: Good manufacturing practice
HGF: Hepatocyte growth factor
IGF-1: Insulin-like growth factor-1
IL-6: Inerleukin-6
MCP-1: Monocyte chemoattractant protein-1
MSCs: Multipotent mesenchymal stromal cells
PL: Platelet lysate
RT: Room temperature
sVCAM-1: Soluble vascular cell adhesion molecule-1
TGF- β1: Transforming growth factor-beta1
VEGF-A: Vascular endothelial growth factor-A
WJ-MSCs: Wharton’s jelly multipotent mesenchymal stromal cells
